# Habitat suitability model for identifying human-wildlife interface and implications for wildlife trade of Sunda pangolin in Borneo

**DOI:** 10.1007/s10661-025-14922-6

**Published:** 2026-01-08

**Authors:** Chrishen R. Gomez, Caroline C. Sartor, David W. Macdonald, Paul J. Johnson, Benoit Goossens, Elisa Panjang, Penny C. Gardner, Nicola K. Abram, Roshan Guharajan, Seth T. Wong, Jaffly Jubili, Jasrin Kuntagil, Siti Nurain Ampuan Acheh, Johny Kissing, Wilvia O. William, Jedediah Brodie, Olga Helmy, Henry Bernard, Ikki Matsuda, Andrew J. Hearn

**Affiliations:** 1 Wildlife Conservation Research Unit, Department of Biology, University of Oxford, Oxford, UK; 2 Organisms and Environment Division, Cardiff School of Biosciences, Cardiff University, Sir Martin Evans Building, Museum Avenue, Cardiff, CF10 3AX UK; 3 Danau Girang Field Centre, c/o Sabah Wildlife Department, 5th Floor, Block B, Wisma MUIS, 88100 Kota Kinabalu, Sabah Malaysia; 4 Pangolin Aware, Lorong Nuri 5, Jalan Labuk, 90000 Sandakan, Sabah Malaysia; 5 Sabah Wildlife Department, Wisma MUIS, Block B, 5th Floor, 88100 Kota Kinabalu, Sabah Malaysia; 6 Department of Ecological Dynamics, Leibniz Institute for Zoo and Wildlife Research, Berlin, 10315 Germany; 7 Division of Biological Sciences, University of Montana, Missoula, MT USA; 8 RSPB Centre for Conservation Science, The Royal Society for the Protection of Birds, The Lodge, Sandy, SG19 2DI UK; 9 Institute for Tropical Biology and Conservation, Universiti Malaysia Sabah, 88999 Kota Kinabalu, Sabah Malaysia; 10 Wildlife Research Center of Kyoto University, 2-24 Tanaka-Sekiden-Cho, Sakyo, Kyoto, 606-8203 Japan; 11 Chubu Institute for Advanced Studies, Chubu University, Kasugai, Aichi Japan; 12 Forest Solutions Malaysia , Sdn. Bhd. F-55-G, 1 Avenue Commercial Centre, Jalan WK, 89108 Kota Marudu, Sabah Malaysia; 13 Panthera Wild Cat Conservation Malaysia C/O Forest Research Centre, Sandakan, Malaysia; 14 Forever Sabah, Kota Kinabalu, Malaysia; 15 Sabah Forestry Department, Sandakan, Sabah Malaysia; 16 Institute of Nature Conservation, Polish Academy of Sciences, Krakow, Poland

**Keywords:** Sunda pangolin, Habitat suitability, Poaching OR Human-wildlife conflict

## Abstract

Pangolins are the most trafficked mammals in the world. Sunda pangolins (*Manis javanica*), in particular, are critically endangered due to their proximity to consumption hotspots and the scale of the globalized illegal trade network. Data on their ecological drivers can inform targeted strategies to cauterize supply lines. We used data from 1455 camera-stations deployed between 2008 and 2024 across a heterogeneous mix of landscapes in Sabah, northern Borneo, to model the geomorphological and anthropogenic drivers of Sunda pangolin distribution. Our most parsimonious logistic regression model included six variables: accessibility to human population (*β* = 0.597, *p* = 0.004), soil cation exchange capacity (*β* = −0.665, *p* = 0.003), soil clay content (*β* = −0.311, *p* = 0.051), soil nitrogen concentration (*β* = 0.9862, *p* = 0.0001), soil bulk density (*β* = 0.43, *p* = 0.143), and topographic position index (*β* = −0.61, *p* = 0.005). The model performed well as evaluated using an out-of-sample test dataset (sensitivity = 0.89, specificity = 0.57, and AUC = 0.73). A high proportion (~ 43%) of rural, human-dominated areas were identified as highly suitable pangolin habitat, but only ~ 15% of these areas are protected. We further confirmed the overlap in highly suitable pangolin habitat and human-occupied land using an independent citizen science dataset of pangolin detections collected between 2019 and 2024 (Boyce index = 0.75). Our results illustrate that Sunda pangolins often live in high-risk areas but also suggest an opportunity to develop community-centered conservation strategies to curb poaching and cauterize supply lines fueling the trade of Sunda pangolins in Southeast Asia.

**Supplementary Information:**

The online version contains supplementary material available at 10.1007/s10661-025-14922-6.

## Introduction

Pangolins have occupied the curiosities of both indigenous communities and western natural historians alike for centuries, most notably through local folklore (Picton, 1988) and early illustrated natural history publications (English School, 1860; Sève, 1761). For Sunda pangolins, the commercial exploitation began in response to their scales being an important ingredient in Traditional Chinese Medicine (TCM) purportedly to promote blood circulation, stimulate lactation, and expel pus. Records from the early twentieth century document an active and intensive harvest destined for China for TCM purposes (Dammerman, 1929). Between 1958 and 1964, Harrisson and Loh (1965) documented 60 tonnes of scales exported from Indonesian Borneo to China via Sarawak, Singapore, and Hong Kong.

Post-globalization, more stringent enforcement through multi-lateral CITES agreements has not succeeded in stemming this flow (Challender et al. 2015). Between August 2000 and July 2019 alone, an estimated 65,000 Sunda pangolins were illegally traded internationally, making up 7% of overall illegal wildlife trade (Challender et al., 2020b). The vast quantities of pangolin constituents needed to supply the traditional Chinese medicine market (Xu et al., 2016) are a leading driver of extinction for all eight species of pangolin (Ellis, 2013). Pangolins are thought to be the most heavily trafficked wild mammal in the world (Aisher, 2016). Exacerbated by their proximity to consumer markets, Sunda pangolins in Southeast Asia are experiencing severe consumptive pressure (Challender et al., 2020a), where numerous patented products continue to use pangolin scales despite the lack of sustainable supply (Hughes, 2021). Globalization and proliferation of transboundary trade routes in Southeast Asia have facilitated the formation of complex supply chains to meet this growing demand.

The Malaysian state of Sabah in northern Borneo was highlighted as a hotspot in the trade of Sunda pangolins when enforcement authorities confiscated 30 tonnes of Sunda pangolin constituents (TRAFFIC, 2019). Since 2017, Sabah has been implicated as playing a crucial role in the smuggling of over 40 tonnes of pangolins between 2017 and 2019. The large quantities of pangolins that move through the illicit market indicate either that poaching networks are becoming more effective and/or that Sunda pangolins are interfacing more frequently with human populations (Chong et al., 2020). This increased human-pangolin intersection may have implications for where the species persists across human-dominated landscapes. At present, the primary strategy for intercepting the sale of pangolins remains reactive, relying on voluntary reporting and information from local communities who interface with pangolins. For combating trade, the International Union for Conservation of Nature and Natural Resources (IUCN) recommends strategies that do not rely solely on enforcement but which also work collaboratively with local communities to dismantle the incentive structures that prop up the illicit networks ( Skinner et al., 2021).

In regions rich in wildlife, like Borneo, habitat for wildlife and humans regularly intersect. Understanding the distribution of Sunda pangolins is key to exploring how this species persists in mixed-used landscapes and can also help guide community engagement. Despite the vast quantities of Sunda pangolins that emerge in the illicit market, quantitative studies of the species in the wild remain scant as the animals are difficult to observe (Chong et al., 2020). The heterogenous mix of landscapes found in Sabah poses a challenge for understanding the habitat niche of Sunda pangolins as they have been observed in a range of habitats including secondary forests, peat swamp forests, wetlands, grasslands, and even monoculture plantations including palm oil and urban gardens (Chong et al., 2020). A previous study has explored habitat associations of Sunda pangolins in natural forest reserves (Panjang et al., 2024), though the species can be found in a wide variety of degraded and mixed habitat types outside forest reserves where they are more likely to interface with local communities. The current study builds on this earlier work by exploring finer-scale habitat predictors and modelling pangolin occurrence across a broader range of human-modified landscapes. This approach allows us to better understand how pangolins interact with environmental and anthropogenic features beyond forest reserves.

The link between an individual’s fitness and their environment is defined by a species’ ecological niche. Habitat suitability models (HSMs) are an operational application of ecological niche as they use environmental variables to predict the absence/presence of a species (Hirzel et al., 2006). Additionally, species interact with both environmental and anthropogenic features at a range of scales relative to their ecological requirements (McGarigal et al., 2016). Thus, the choice of habitat and consequently, the core distribution of a species is a scale-dependent process. To understand accurately the relationship between Sunda pangolins and habitat suitability predictors, this modelling framework identifies the scale at which key variables drive a species’ habitat niche, particularly in complex, human-modified landscapes. In this study, we use a logistic regression modelling framework to model the detection of Sunda pangolins from a camera-trap dataset for Sunda pangolins in Sabah. Specifically, our goals were to (1) identify key environmental variables that explain the current distribution of Sunda pangolins in Sabah, (2) predict the distribution of Sunda pangolins in areas of Sabah outside our study area, and (3) map risk zones for human-wildlife conflict in Sabah. These insights will help us understand how rare and cryptic mammals persist in fragmented tropical environments.

## Methods

### Overview

To investigate the drivers of Sunda pangolin distribution in Sabah, we compiled a camera-trap dataset spread throughout the state. Data were collected over 16 years (2008–2024) across a wide variety of landscape and habitat types. We used a multi-scale resource selection function to identify key variables that explain the distribution of pangolins using binary absence and presence records. We ranked and selected the best model using the Akaike Information Criterion (AIC). We predicted habitat suitability across Sabah using this model. We quantified the area of suitable habitat that is currently found inside and outside protected areas.

### Camera-trapping

The dataset comprised several independent camera-trap surveys that were designed following best practices for detecting small to medium-sized mammals in tropical systems. We used several models of professional scientific camera-traps Reconyx Hyperfire 2, Reconyx HC 500, Pantheracam V6, and Bushnell Trophy Cam that are regularly used for studying cryptic species in tropical systems. Camera-traps were deployed on tree barks 40–60 cm from the ground. Each camera was set to capture three images per detection with no delay between triggers and no sleep delay before retriggering. The camera-traps used infrared flash in low-light conditions to minimize disturbance to nocturnal species. Each survey was carried out for a minimum of 60 days.

### Data preparation

**Fig. 1:**
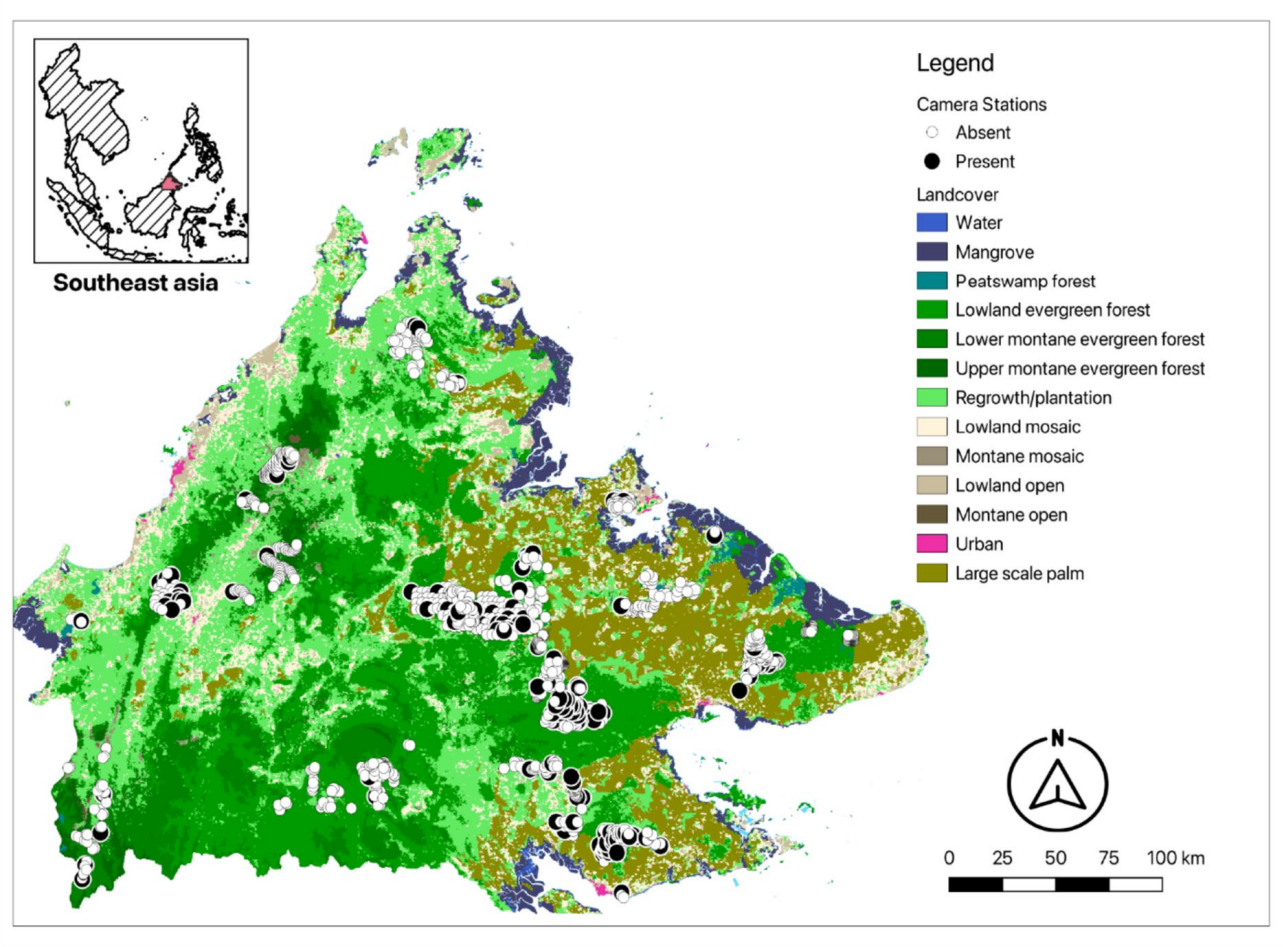
Distribution of the 1455 camera-stations used in the analysis. White and black points on the map denote non-detections and detections, respectively. Land use categories were derived from a Southeast Asia wide landcover map at 250 m resolution (Miettinen et al., 2016)

The dataset was compiled from a network of seven research groups operating a total of 1555 camera-stations across Sabah (Fig. 1). The sites were distributed across a variety of habitat types, both inside and outside forest areas (Table 1). We filtered camera-stations that operated for a minimum of 30 days and removed duplicated camera-stations found within a circular buffer of 500 m to minimize spatial autocorrelation. To ensure camera-trapping surveys were suitably designed and deployed to detect Sunda pangolins, we removed any surveys which completely failed to detect any Sunda pangolins. After removing camera-trapping grids that failed to detect Sunda pangolins, we were left with a total of 1455 camera-stations. From this total, 138 had recorded Sunda pangolins. Sunda pangolins are rare and therefore often result in datasets with a large proportion of zero values. This form of zero-inflation causes problems for statistical inference as the data does not readily fit standard distributions (Martin et al., 2005), which can result in poor performance of models (Barbet-Massin et al., 2012). Generalized linear models perform poorly at low prevalence (< 0.1) and achieve optimal predictive performance when prevalence is higher than 0.2 (Barbet-Massin et al., 2012). To address the low prevalence in our dataset (< 0.1) and our inability to distinguish between false and true absences, we bootstrapped the absence records ten times to create ten datasets of equal numbers of absences and presences. Absences were sampled from each survey area to match the number of presences recorded in that survey. Therefore, we produced ten datasets with equal absences and presences (*n* = 276). We partitioned the total dataset for training and testing following a 4:1 partitioning ratio (Rana et al., 2024).

**Table 1:** Distribution of camera-stations used in analysis by landcover category. Evergreen forest includes lowland, lower montane and upper montane forest types. While mosaic includes lowland, montane and lowland open

Landcover Category	Number of camera-stations	Percentage of total cameras (%)
Evergreen forest	1080	74.2
Regrowth/plantation	248	17.04
Peat swamp forest	14	0.96
Mangrove	20	1.3
Mosaic	72	4.9
Palm oil plantation	22	1.5

### Spatial predictors

Chong et al. (2020) noted that little was understood at the time about the factors influencing a pangolin’s choice of habitat. We fit a model using predictors that have been previously used to model the spatial distribution of wild animals in the fragmented and heterogeneous landscapes on Borneo and Southeast Asia (Sartor et al., 2024). We used a total of 27 spatial predictors thought to be relevant to Sunda pangolin ecology (Table S5). As our camera-trap data spanned a total of 14 years (2008–2024) (Table S1), we matched the values of spatial predictors to the corresponding year of the detection. In so doing, we removed the effect of differential years of detection due to changes to the landscape matrix.

### Multi-scale optimization and variable selection

All analysis, unless stated otherwise, was performed in R v4.4.2 (R Core Team, 2024). To identify the optimized scale for each predictor, we calculated the focal mean of each predictor variable at seven spatial scales (0.25 km, 0.5 km, 1 km, 2 km, 4 km, 8 km, 16 km) following the known home ranges of Sunda pangolins (Gray et al., 2023). For this analysis, each layer was recalculated based on a Gaussian Kernel with a radius equal to the desired scale and a standard deviation equal to half of the target scale using ee.Kernel.gaussian function in Google Earth Engine. Thus, this function applied spatial smoothing in the rasters, where the pixel value is weighted based on its spatial proximity to the central pixel, following a normal distribution. This smoothing allows us to keep the general spatial trends of the original rasters. We extracted raster values around each camera-trap location for each predictor at each scale. An animal’s relationship with its habitat is known to be functionally heterogeneous, resulting in a scale-dependent relationship to individual habitat components. We performed univariate scaling analysis to select the scale with the strongest likelihood of predicting the dataset. Univariate scaling is a known method for identifying the optimal scale in habitat relationship modelling (McGarigal et al., 2016; Wasserman et al., 2012). After selecting the optimal scale for each spatial predictor, we calculated Pearsons’ pairwise correlations (Figure S1). If two variables were correlated |r|> 0.7, we retained the variable with the lower AIC from the univariate GLM. We also calculated the variance inflation factor (VIF) and removed predictors that were collinear (vif > 5). Our final set of candidate variables was reduced from 27 to 14 predictors individually optimized for scale. The above methods for scale optimization and selection were performed based on methods in (Chiaverini et al., 2023).

### Multivariate modelling and model evaluation

With species detection as a response, we fitted a logistic regression model with a binomial distribution. We identified the best predictor combination fitting a model for every possible combination of predictors using the *dredge* function in *MuMin* v1.48.4 (Barton & Barton, 2015). We ranked the models by calculating the Akaike Information Criterion (AIC) and selected the best model (ΔAIC = 0). We evaluated the predictive performance of the best model using the test dataset. To identify the optimal classification for probability predictions, we tested 30 threshold values between 0 and 1 and calculated the AUC and Kappa score from a confusion matrix of observed and predicted values. We selected the threshold with the highest AUC score. The scale optimization, modelling, and evaluation steps were repeated iteratively for all ten bootstrapped datasets (Table S3). This resulted in ten such models. For each of these, an optimal threshold was selected based on AUC and Cohen’s kappa scores. The final model used was the one with the highest predictive scores.

### Model validation with independent dataset

We used data collected by the Sabah Wildlife Department on Sunda pangolin rescues as an independent dataset. Rescue incidences were predominantly opportunistic encounters of Sunda pangolins by various groups of people across different landscapes, who then informed local NGOs or state authorities. This dataset consisted of 149 spatially explicit events, which were used to validate the predictions from our model (Figure S5). We evaluated model performance using the Boyce index (Boyce et al., 2002), which has been shown to correlate well with AUC predictions for habitat suitability models (Hirzel et al., 2006). This test was performed to measure the overall predictive power of our model for estimating the risk of overlap between Sunda pangolin habitat and human populations.

### Gap analysis

To understand the level of protection and potential risks associated with Sunda pangolin habitat, we obtained data on protected areas in Sabah following Panjang et al. (2024). Sabah’s Totally Protected Area (TPA) network includes Wildlife Sanctuary, Class I Protection Forest Reserve, Class VI Virgin Jungle Reserve, Class VII, which are equivalent to IUCN Category 1a (strict nature reserves), but also Wildlife Reserve Park and Wildlife Conservation Area, which are classed as IUCN Category IV (habitat or species management area). We first classified the likelihood of habitat suitability into four categories (*low*, *medium low*, *medium high*,and *high*)*.* As the output of our model prediction is a back transformed probability between 0 and 1, each suitability category was discretised using four bins of equal width. We then used the *zonal statistics* function in QGIS (*QGIS 3.34*) and measured the total area of each category of habitat suitability within protected areas.

To quantify the risk of conflict in rural landscapes used by communities, we calculated the proportion of suitable habitat for three land use classes. These data were obtained from a social High Conservation Screening Assessment for Sabah (Abram et al., 2025). These data were developed through on-screen digitizing (in ArcGIS 10.4.1) of high resolution (1.5 m) satellite imagery (SPOT 5 & 6) for the years 2014 and 2015. The three land use classes included: (1) Wet paddy areas, mainly located in western Sabah; (2) Areas dominated by oil palm smallholdings (but also including dispersed homesteads within these landscapes), largely associated with the eastern half of Sabah; and (3) A generalized “community mosaic area” layer (largely associated with the western half of Sabah) that included mixed land uses important to local communities, small-scale farming (rubber, coconut, hill rice, vegetables, fruit), shifting cultivation, orchards, forest patches, some small scattered oil palm smallholdings (not dominant in the landscape), dispersed homesteads, and small villages.

## Results

### Variable scale selection after correlation

After removing correlated and collinear predictors, we had a final set of 14 spatial predictors (Table S4). These revealed 26 models ranked as having strong or moderate support (ΔAICc < 2) (Burnham & Anderson, 2002). The top ranked model incorporated six predictors (Table 2). Topographic position index, soil nitrogen concentration, and soil cation exchange capacity had the strongest evidence for effects. There was weaker evidence for a positive effect of soil bulk density.

**Table 2:** Coefficients of predictors selected in the top ranked logistic regression model with lowest AICc value with presence/absence as a response. (* denotes statistical significance)

	***Estimate***	***Std. error***	***z value***	***Pr (* >*|z|)***
(Intercept)	−0.0563	0.1566	−0.3595	0.7193
Accessibility to Human Pop. Index_100m	0.5967	0.2053	2.9065	0.0037*
Soil Bulk Density_100m	0.4301	0.2939	1.463	0.1435
Soil Cation Exchange Capacity_16000m	−0.6648	0.2241	−2.9661	0.003*
Clay content_100m	−0.3105	0.1591	−1.9521	0.0509*
Soil Nitrogen_8000m	0.9862	0.2591	3.8065	0.0001*
Topographic Position index_16000m	−0.6098	0.1745	−3.4944	0.0005*

### Model selection

The model was tested using a confusion matrix between the observed and predicted responses. The optimal threshold for maximizing prediction strength was 0.35, which yielded a predictive power of 0.89 sensitivity and 0.57 specificity (AUC = 0.73, TSS = 0.46).

### Response curves of predictor variables

**Fig. 2:**
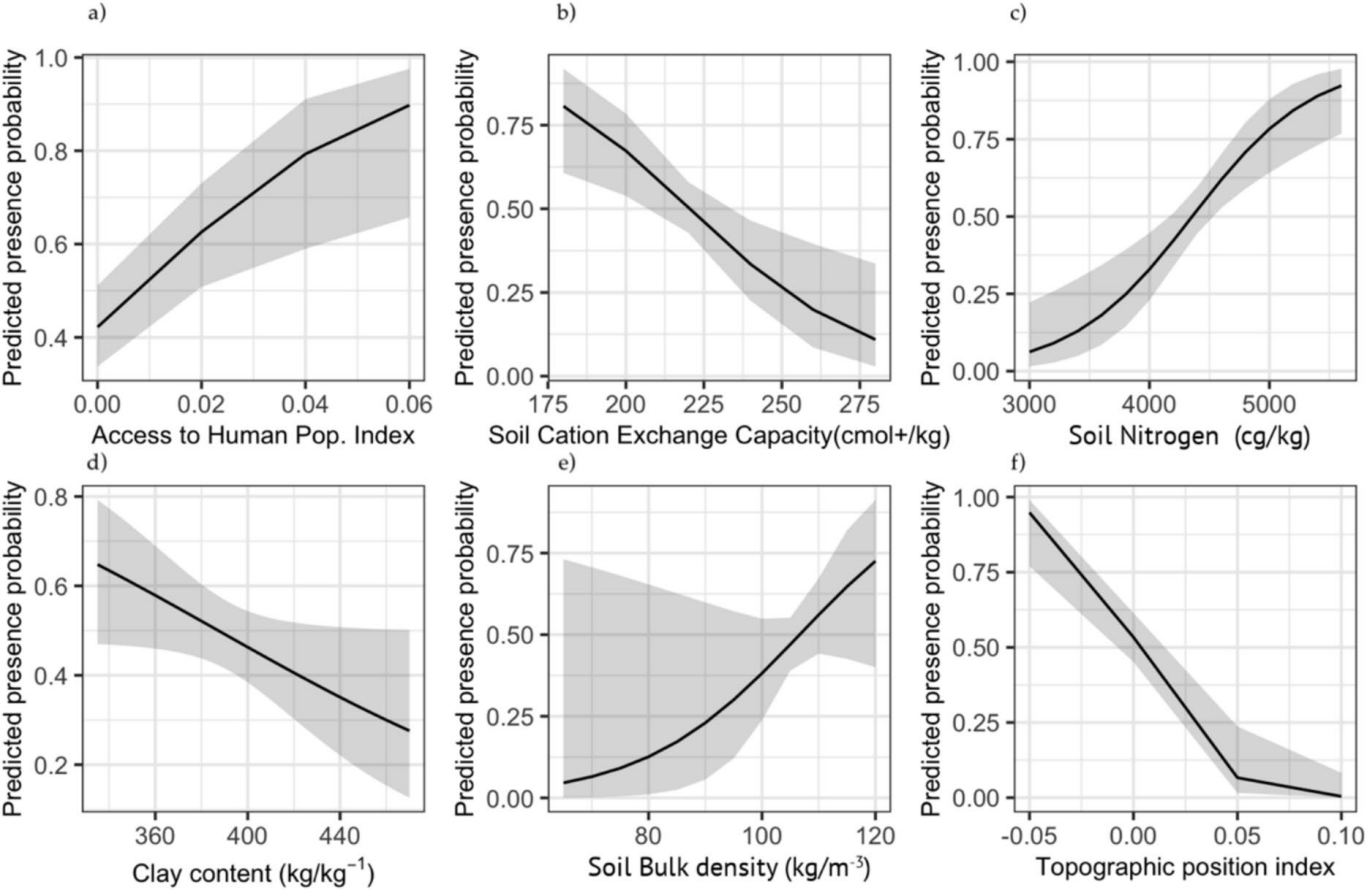
**a**–**f** Scaled response curves for the six variables in final model selected reflecting relationship between each predictor and the probability of detecting Sunda pangolins based on presence-absence records

We identified a strong positive relationship between Sunda pangolins and accessibility to human populations (Fig. 2a). Soil type predictors were widely represented among the final list of six candidate variables indicating a strong association between pangolin occurrences and soil chemistry. Specifically, Sunda pangolins had a strong negative relationship with carbon exchange capacity (Fig. 2b) and a strong positive relationship with soil nitrogen concentration (Fig. 2c). Sunda pangolins had a negative association with clay type soil (*p* < 0.05) (Fig. 2d) and a weak positive association with soil bulk density (Fig. 2e). Topographic position index (TPI) relates to the position of a pixel relative to its surrounding landscape, with negative values indicating valleys or depressions. Sunda pangolins were strongly associated with negative TPI values (Fig. 2f).

The independent Sunda pangolin rescue dataset was strongly correlated with the overall habitat suitability model predictions (Boyce Index = 0.75), indicating strong support for the model. Specifically, the dataset validates the model’s ability to predict areas where humans and Sunda pangolins are likely to interact. We found a positively linear relationship between habitat suitability and the ratio between predicted and expected values for our data (Figure S2).

### Gap analysis and conflict risk

**Fig. 3:**
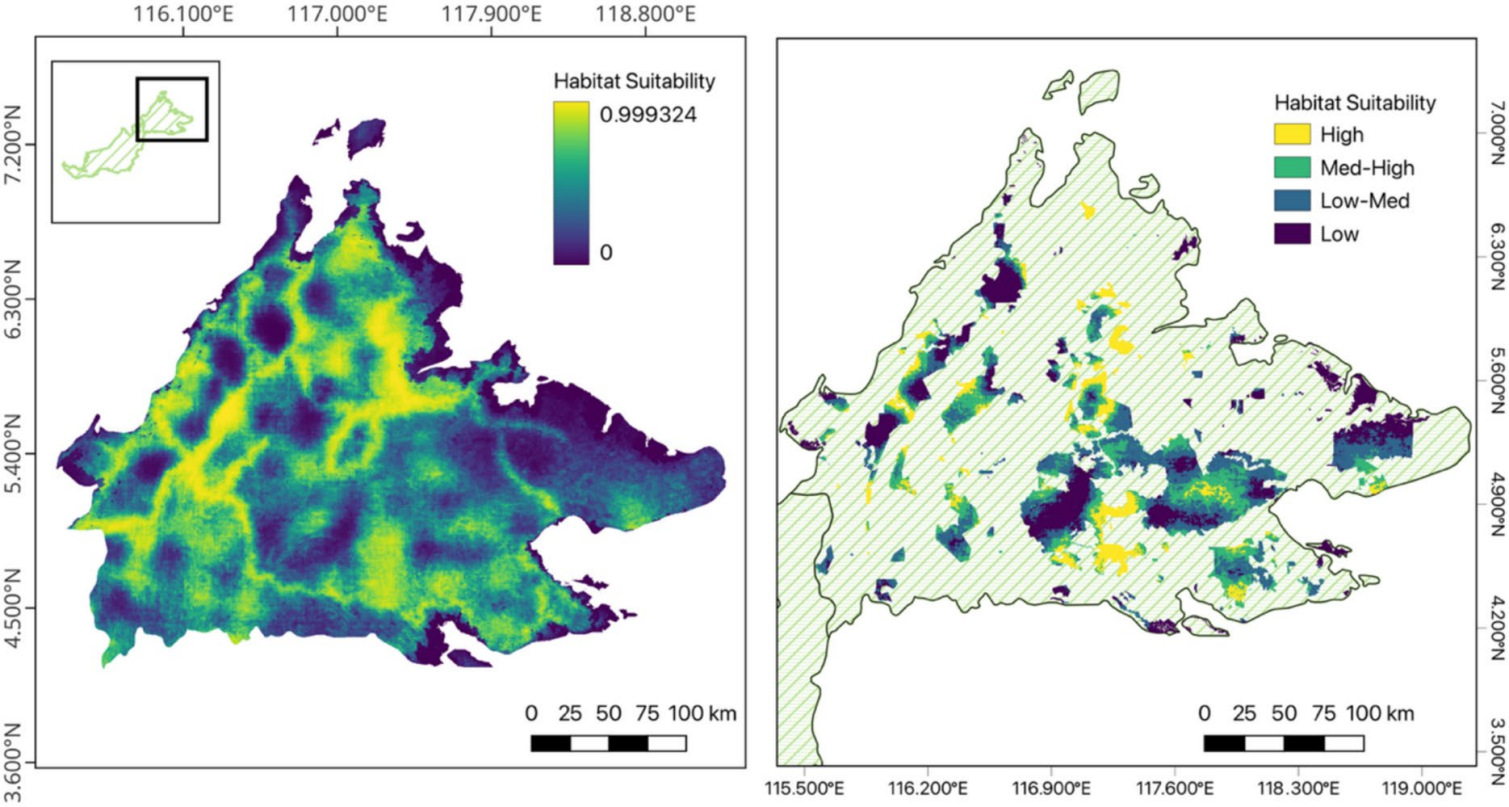
**a** Habitat suitability prediction across Sabah using our best ranked model, with 0 representing lowest predicted suitability and 1 representing highest predicted suitability; and (**b**) A categorical habitat suitability prediction binned in four quartiles where Low (x < 0.25), Low-Med (0.25 ≤ x < 0.5), Med-High (0.5 ≤ x < 0.75), and High (0.75 ≤ x < 1) within Sabah’s protected area network. Suitability was calculated using values of scale-optimized selected predictors in Sabah (Figure S4)

**Fig. 4:**
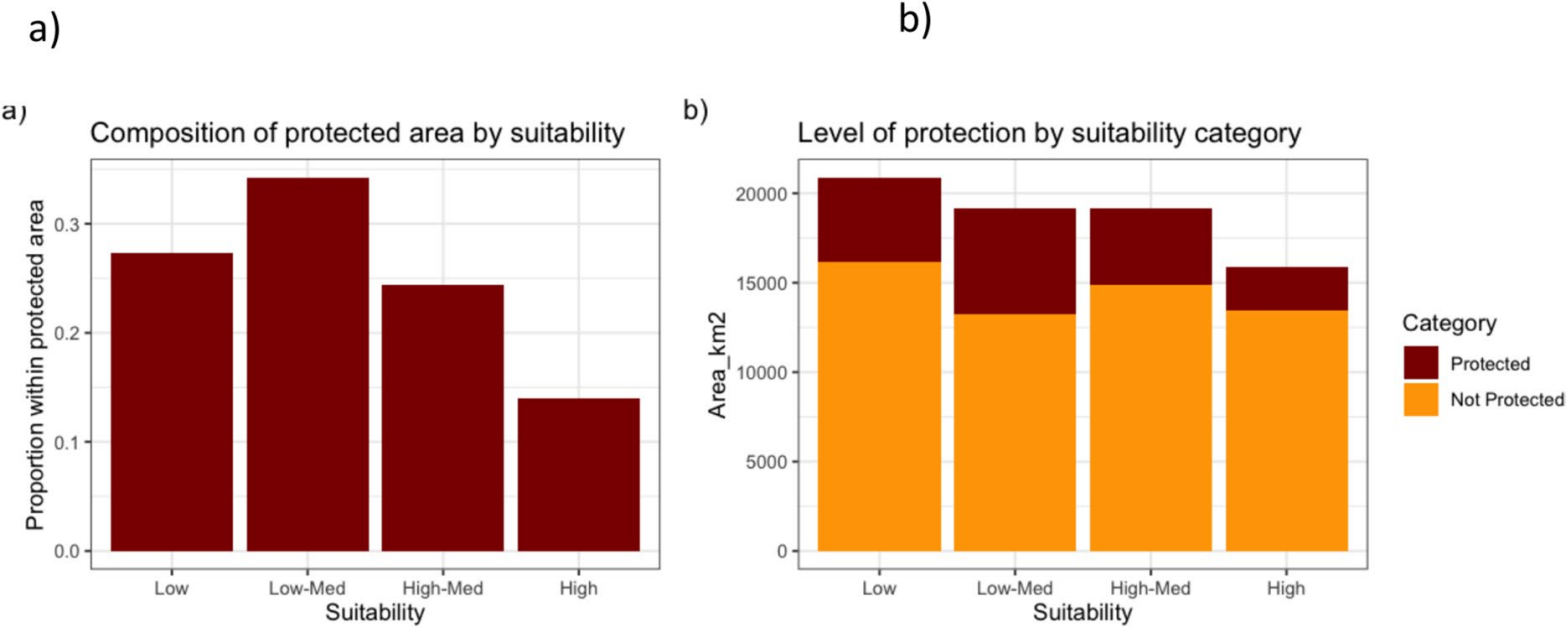
**a** Proportion of each suitability category found within protected area. **b** Total extent (km^2^) of suitable area that are protected and not protected in Sabah

After creating 4 classes of suitability, we found that habitat within the highest suitability class was also the least protected by area (15.3%) (Fig. 3). The suitability class that was most protected was low-medium (~ 31%). Within totally protected areas, we found that the largest portion of land was of low (0.27) and medium low (0.34) suitability, while high suitability habitat represented the smallest proportion of totally protected area (0.14) as visualized in Fig. 4.

**Fig. 5:**
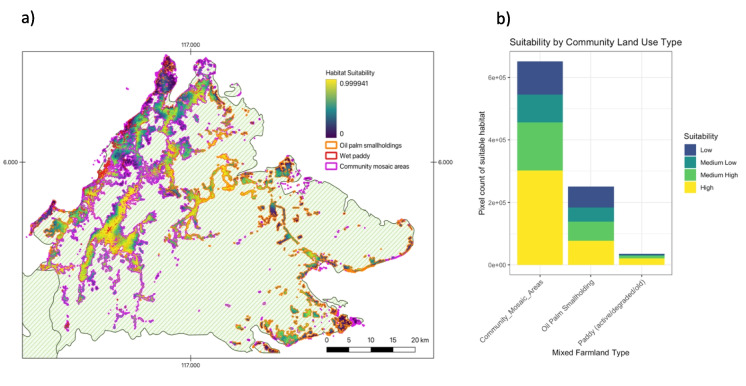
**a** Habitat suitability index as distributed across community land use areas across Sabah. **b** Proportion of habitat suitability across three community land use types (paddy, areas dominated with oil palm smallholdings, and a more generalized community mosaic area)

Spatial analysis of human use of rural land showed high overlap with highly suitable habitat (Fig. 5a). Cumulatively, rural land used by humans was dominated by highly suitable habitat (42.6%), followed by medium high (23.9%), low (18.8%), and medium low suitability (14.7%) for pangolins (Fig. 5b). Across all community-associated land use types, highly suitable habitat comprised the largest proportion of land. The community land use type that had the highest proportion of highly suitable habitat was paddy (59.2%), smallholding mixed areas (54.1%), and agroforestry (42.3%) (Fig. 5b). Oil palm smallholdings constituted the most balanced proportion of pangolin habitat suitability (32% high, 24% medium high, 17% medium low, and 25% low).

## Discussion

As with Panjang et al. (2024), we report a strong relationship between Sunda pangolin distribution and soil composition. In their model, soil associations represented 21.2% of overall explanatory power but did not resolve specific geomorphological properties of those associations. Our analysis benefited from substantial progress in machine learning classifiers for characterizing soil types, and access to greater detail across Sabah, allowing us to reveal the specific drivers of this relationship (Hengl et al., 2017). Sunda pangolins are specialist myrmecophages, feeding primarily on ants and termites (Cabana & Tay, 2020; Zhang et al., 2021). Termites feed primarily on plant material in varying stages of decay while ants can be omnivorous, opportunistic feeders or herbivores. Both groups are major ecosystem engineers and affect soil properties and resource availability for other taxa by nest building (Luke et al., 2014). Ant and termite distribution are also strongly influenced by fine-scale environmental variables such as slope, leaf litter quality, humus depth, grass, and bare ground cover (Luke et al., 2014).

Concentration of nitrogen (N) in soil presented the strongest effect in our model, with likelihood of pangolin detection increasing with concentration. In terrestrial systems, increase in N deposition is linked to increase in net primary production (NPP). While less so for termites, ants are well represented in disturbed, human-dominated landscapes such as logged forest, due to the higher temperatures, availability of nest-sites, and simpler surface structures that encourage foraging (Luke et al., 2014). Termite abundances are, however, higher in old-growth forest, suggesting an ability for Sunda pangolins to persist primarily on ant species that are more abundant in degraded and human-dominated landscapes.

We demonstrate a significant negative relationship between pangolin occurrence and the topographic position index, which we interpret as a preference for valleys, ditches, and lower slopes. TPI is an important predictor in ecology as it is a composite measure of slope, elevation, and aspect. Negative TPI is generally associated with higher soil organic carbon due to an accumulation of water and soil erosion particles for upper slopes but varies in the context of vegetation composition at upper slopes (Seibert et al., 2007). Additionally, occurrences of Sunda pangolin were weakly but positively associated with soil bulk density, translating to a preference for more compact soil. Studies on Chinese pangolins (*Manis pentadactyla*) showed that they preferentially select site burrows in soils with higher bulk density, pH, and nitrates (Sun et al., 2024).

The habitat suitability model we present offers insights into the hotspots for Sunda pangolin poaching and illegal trade. Areas of high suitability will likely be source points that supply the globalized illicit network. In this paper, we use data provided by Sabah Wildlife Department, as an independent test of predictions made by the model. These predictions can be used to develop dynamic conservation strategies that target specific communities through a co-design process. The IUCN explicitly recommends community involvement as a pre-requisite to enforcement in the context of isolating the supply and demand for illegal wildlife products (Skinner et al., 2021).

The analysis of community use land indicates a high overlap of suitable habitat in community areas used for commercial purposes. These findings highlight the central importance communities play in promoting co-existence with Sunda pangolins. The positive Boyce index scores (0.75) indicate a strong correlation between areas of predicted high suitability and that of recorded cases of interaction between human settlements and Sunda pangolins. While our model was trained solely on camera-trap data collected in the field, the validation with citizen science records affirms the overlap between Sunda pangolin habitat preferences and human settlements. It is also noteworthy that the citizen science dataset recorded a comparable number of “detections” (*n* = 139) in 5 years (2019–2024) as the camera-trap dataset between 2008 and 2021 (*n* = 138). The high degree of overlapping area highlights the importance of addressing anthropogenic specific threats to pangolins, such as poaching and the prevalence of feral dogs and road collisions.

Despite the tight link with human-dominated landscapes, only one of the six selected variables in the final model is linked directly to human densities; the remaining five are measures of landscape geomorphology, suggesting shared geomorphological drivers of both Sunda pangolin distribution and human settlement and agricultural expansion. On Borneo, the expansion of human-dominated landscapes between 1973 and 2015 was driven primarily by socioeconomic opportunities linked to the export of timber and palm oil (Gaveau et al., 2014).

In Sabah, we show that Sunda pangolins are associated with habitat outside protected areas where they are more likely to be encountered by humans. This might be explained by the non-random distribution of protected areas. Protected areas are often delineated using a suite of bio-physical and socioeconomic factors including elevation, population density, proximity to roads and agricultural potential in lieu of a systematic conservation strategy (Joppa & Pfaff, 2009). In other studies conducted in Sabah, protected areas were shown to conserve only ~ 40% of areas with high species richness (Scriven et al., 2020) and did not meaningfully protect habitats that compete with other land uses (Macdonald et al., 2024). Importantly, however, the current matrix of protected areas does protect core habitat for wild felids (Chiaverini et al., 2025) and butterflies (Scriven et al., 2020), which generally serve as good biodiversity indicators (Tossens et al., 2025).

These findings indicate that pangolins are driven by an unusual set of ecological drivers that necessitate innovative conservation strategies to address the infelicitous overlap between their contemporary habitat selection and the elevated risks posed by proximity to human settlement. It is possible that the decades of commercial exploitation of Sunda pangolins could have resulted in the near extirpation of Sunda pangolins in natural forested habitats. While plausible, habitat generalism has been a marked feature of Sunda pangolin behavior from the earliest natural history records where they were regarded as “common in the Malay peninsula” (Ridley, 1926).

The lower detections of Sunda pangolins in natural forests could also be explained by reduced detection probability leading to false negatives in forest habitat (Martin et al., 2005). Statistical approaches that disentangle detection probabilities from true presence/absence will solve this problem but require additional information such as repeated detections or non-detections over the survey period. Several surveys within our dataset lacked sequential data on detection windows that are needed to estimate detection probability. Our inability to ascertain detection probabilities meant that we could not determine the proportion of false zeros in our dataset. The choice to cull a large number of zero (absence) records in this study was designed to improve model performance by narrowing the ratio between absences and presences. We bootstrapped these data to evaluate the robustness of our final selected model. While the models did not have perfect consensus, there was some reassuring consistency: soil variables which featured prominently in our final model were also a dominant feature in the models of all ten bootstrapped datasets (Table S3). However, we strongly encourage future studies to gather data that will enable reliable detection probability estimation for each camera. This, no doubt, will require finer-scale temporal resolution of the data, but we consider this a worthwhile pursuit in handling variable detection probabilities for compiled databases.

## Conclusion

The habitat suitability model we present in this paper provides a powerful and useful tool for predicting hotspots of high intersection between the occurrence of Sunda pangolin and local communities. As the most illegally trafficked mammal in the world, identifying regions where pangolins and humans interact regularly can help identify “source points” of the illegal supply chain. Our model, therefore, facilitates the deployment of conservation strategies to affected communities directly, in line with IUCN illegal wildlife trade guidelines. In this paper, we also demonstrate effective methodology for integrating citizen science in scientific analysis of species occurrence. The paper leverages the availability of the independent dataset to test and validate the predictions of the habitat suitability model. The independent citizen science dataset we present highlights the benefits of engaging local communities in a collaborative manner with state wildlife agencies. In our model, we also highlight the inadequacy of the current configuration of protected areas in Sabah for protecting Sunda pangolin habitat. Therefore, we emphasize the importance of community-first strategies that incentivize communities living near and around Sunda pangolin habitat to be involved in their protection.

## Supplementary Information

Below is the link to the electronic supplementary material.

ESM 1 Supplementary Material 1 (DOCX 1.76 MB)

## Data Availability

No datasets were generated or analysed during the current study.
