# Supplementary material for: Habitat suitability model for identifying human-wildlife interface and implications for wildlife trade of Sunda pangolin in Borneo
Source: Environ Monit Assess. 2026 Jan 8;198(2):108. doi: 10.1007/s10661-025-14922-6 (PMC12783250; doi:10.1007/s10661-025-14922-6)
Supplement: Supplementary file 1 — Supplementary Material 1 (DOCX 1.76 MB) [file 10661_2025_14922_MOESM1_ESM.docx]

# **Habitat suitability model for identifying human-wildlife interface and implications for wildlife trade of Sunda pangolin in Borneo**

Supplementary figures and tables

Table S1: Summary of datasets and sources

| **Dataset** | **Research group** | **Trap effort** | **Total Cameras** | **Trap night per camera** | **Year of Survey** | **Publication** |
| --- | --- | --- | --- | --- | --- | --- |
| 1 | Hearn et al. | 24548 | 199 | 123.4 | 2021-2024 | Unpublished |
| 2 | Deere et al. | 1048 | 25 | 41.9 | 2015 | Deere et al. 2020 |
| 3 | Guharajan et al. | 10195 | 125 | 81.6 | 2018 | Guharajan et al. 2023 |
| 5 | Gardner et al. | 45964 | 424 | 108.4 | 2011-2013 | Gardner et al. 2019 |
| 6 | Bernard et al. | NA | 50 | NA | 2017-2023 | Bernard et al. 2022 |
| 7 | Haysom et al. | 4550 | 50 | 91.0 | 2017-2019 | Haysom et al., 2021 |
| 8 | Hearn et al. | 47469 | 449 | 105.7 | 2013 | Hearn et al. 2019 |
| 9 | Brodie et al. | 6926 | 46 | 150.6 | 2012 | Brodie et al., 2012 |
| 10 | Hearn et al. | 10857 | 73 | 148.7 | 2021 | Unpublished |
| 11 | William et al. | 2017 | 87 | 23.2 | 2018 | Unpublished |


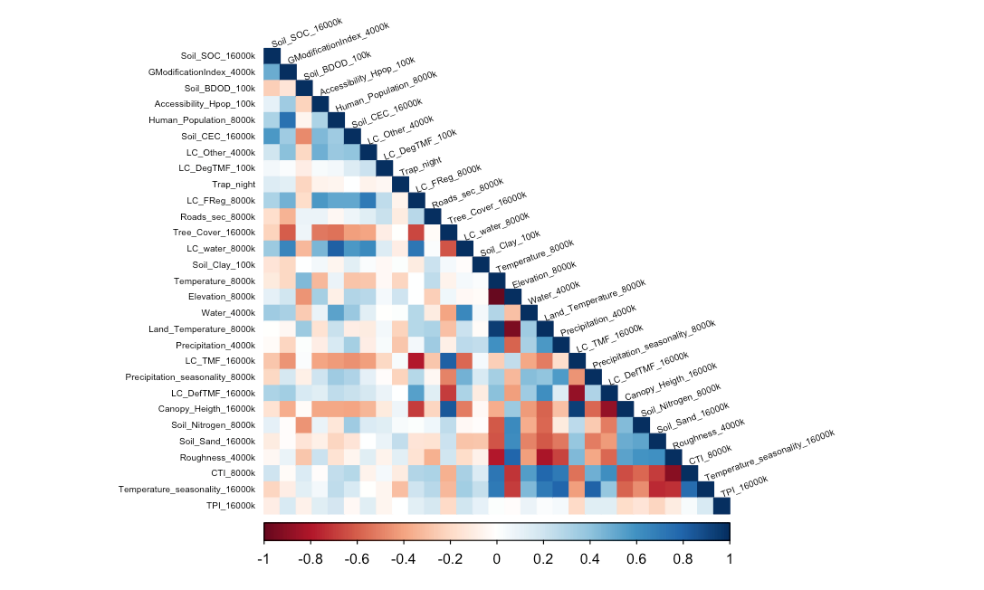


Figure S1: Correlation plot for 30 scaled spatial predictors

Table S2 : Total extent (km**^2^**) of four habitat suitability classes (low, low medium, medium high and high), along with their area (km**^2^**), percentage (%), and proportion within Sabah’s protected areas.

| Habitat Suitability Category | Total extent of habitat area in Sabah (km^2^) | Extent of habitat protected (km^2^) | Percentage of habitat class protected (%) | Proportion of habitat class protected |
| --- | --- | --- | --- | --- |
| Low | 20,887 | 4,737 | 22.7 | 0.227 |
| Low Medium | 19,145 | 5,934 | 30.9 | 0.31 |
| Medium High | 19,147 | 4,236 | 22.1 | 0.22 |
| High | 15,859 | 2,425 | 15.3 | 0.15 |


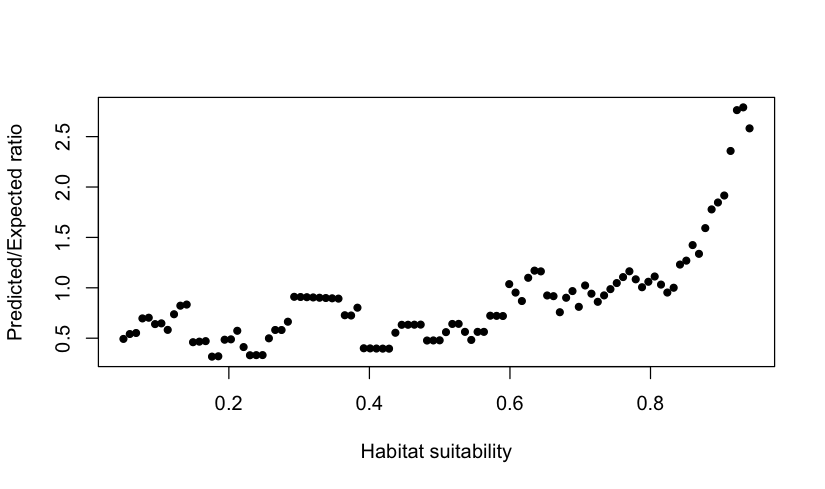


Figure S2: Positive relationship between habitat suitability and ratio between predicted and expected values when calculating Boyce Index using independent rescue dataset.

Table S3: Model variable selection and performance results from bootstrapping absence data. Best model was selected with lowest AUC and highest Kappa score.

| **Model summary** | | | | **Model performance** | | | |
| --- | --- | --- | --- | --- | --- | --- | --- |
| **Randomisation** | **Selected vars** | **Coefficient** | **p** | **Sensitivity** | **Specificity** | **Kappa** | **AUC** |
| 222 | (Intercept) | 0.89 | 0.01 | 0.61 | 0.79 | 0.39 | 0.75 |
|  | Accessibility_Hpop_100k | 16.93 | 0.10 |  |  |  |  |
|  | Precipitation_seasonality_8000k | -0.05 | 0.01 |  |  |  |  |
|  | TPI_16000k | -41.23 | 0.00 |  |  |  |  |
|  | Water_250k | -0.16 | 0.22 |  |  |  |  |
| 333 | (Intercept) | -4.06 | 0.57 | 0.89 | 0.57 | 0.46 | 0.73 |
|  | Accessibility_Hpop_100k | 41.45 | 0.00 |  |  |  |  |
|  | Soil_BDOD_100k | 0.07 | 0.14 |  |  |  |  |
|  | Soil_CEC_16000k | -0.04 | 0.00 |  |  |  |  |
|  | Soil_Clay_100k | -0.01 | 0.05 |  |  |  |  |
|  | Soil_Nitrogen_8000k | 0.00 | 0.00 |  |  |  |  |
|  | TPI_16000k | -55.54 | 0.00 |  |  |  |  |
| 444 | (Intercept) | -0.73 | 0.13 | 0.68 | 0.61 | 0.29 | 0.67 |
|  | Accessibility_Hpop_100k | 30.94 | 0.02 |  |  |  |  |
|  | GModificationIndex_500k | 9.79 | 0.00 |  |  |  |  |
|  | LC_DefTMF_16000k | -3.03 | 0.00 |  |  |  |  |
|  | LC_water_4000k | -20.86 | 0.03 |  |  |  |  |
|  | TPI_16000k | -55.45 | 4.55E-05 |  |  |  |  |
| 555 | (Intercept) | -24.07 | 0.00 | 0.32 | 0.82 | 0.14 | 0.55 |
|  | Accessibility_Hpop_100k | 23.71 | 0.05 |  |  |  |  |
|  | Soil_BDOD_100k | 0.07 | 0.10 |  |  |  |  |
|  | Soil_Nitrogen_8000k | 0.00 | 0.02 |  |  |  |  |
|  | Soil_Sand_16000k | 0.04 | 0.01 |  |  |  |  |
|  | TPI_8000k | -9.50 | 0.03 |  |  |  |  |
| 666 | (Intercept) | -6.50 | 0.23 | 0.5 | 0.79 | 0.29 | 0.64 |
|  | LC_Other_4000k | -12.04 | 0.13 |  |  |  |  |
|  | Soil_BDOD_16000k | -0.08 | 0.03 |  |  |  |  |
|  | Soil_Sand_16000k | 0.05 | 0.00 |  |  |  |  |
|  | TPI_16000k | -47.84 | 0.00 |  |  |  |  |
| 777 | (Intercept) | -11.20 | 0.07 | 0.71 | 0.43 | 0.14 | 0.52 |
|  | Precipitation_seasonality_16000k | -0.03 | 0.09 |  |  |  |  |
|  | Roughness_250k | 0.01 | 0.03 |  |  |  |  |
|  | Soil_BDOD_250k | 0.07 | 0.12 |  |  |  |  |
|  | Soil_Nitrogen_8000k | 0.00 | 0.05 |  |  |  |  |
|  | TPI_16000k | -25.95 | 0.08 |  |  |  |  |
|  | Water_250k | -0.14 | 0.25 |  |  |  |  |
| 888 | (Intercept) | -2.70 | 0.08 | 0.93 | 0.29 | 0.21 | 0.64 |
|  | LC_FReg_100k | -1.99 | 0.05 |  |  |  |  |
|  | Precipitation_seasonality_8000k | -0.05 | 0.01 |  |  |  |  |
|  | Soil_Nitrogen_8000k | 0.00 | 0.01 |  |  |  |  |
|  | TPI_16000k | -24.82 | 0.06 |  |  |  |  |
| 999 | (Intercept) | 1.89 | 0.43 | 0.25 | 1 | 0.25 | 0.64 |
|  | Accessibility_Hpop_16000k | 44.65 | 0.02 |  |  |  |  |
|  | Precipitation_seasonality_4000k | -0.05 | 0.02 |  |  |  |  |
|  | Roads_sec_4000k | -7.70 | 0.03 |  |  |  |  |
|  | Soil_CEC_16000k | -0.03 | 0.02 |  |  |  |  |
|  | Soil_Nitrogen_8000k | 0.00 | 0.00 |  |  |  |  |
|  | TPI_16000k | -35.55 | 0.02 |  |  |  |  |
|  | Water_250k | -0.18 | 0.20 |  |  |  |  |
| 101 | (Intercept) | -12.72 | 0.04 | 0.71 | 0.54 | 0.25 | 0.61 |
|  | Accessibility_Hpop_2000k | 24.70 | 0.03 |  |  |  |  |
|  | LC_water_500k | -13.19 | 0.05 |  |  |  |  |
|  | Soil_BDOD_8000k | 0.07 | 0.11 |  |  |  |  |
|  | Soil_Nitrogen_4000k | 0.00 | 0.00 |  |  |  |  |
|  | TPI_16000k | -35.51 | 0.01 |  |  |  |  |
| 102 | (Intercept) | -13.82 | 0.01 | 0.54 | 0.61 | 0.14 | 0.53 |
|  | Canopy_Height_4000k | 0.07 | 0.02 |  |  |  |  |
|  | LC_Other_16000k | 20.59 | 0.01 |  |  |  |  |
|  | LC_water_500k | -12.35 | 0.09 |  |  |  |  |
|  | Soil_BDOD_250k | 0.07 | 0.08 |  |  |  |  |
|  | Soil_Nitrogen_8000k | 0.00 | 0.02 |  |  |  |  |
|  | TPI_16000k | -25.00 | 0.08 |  |  |  |  |


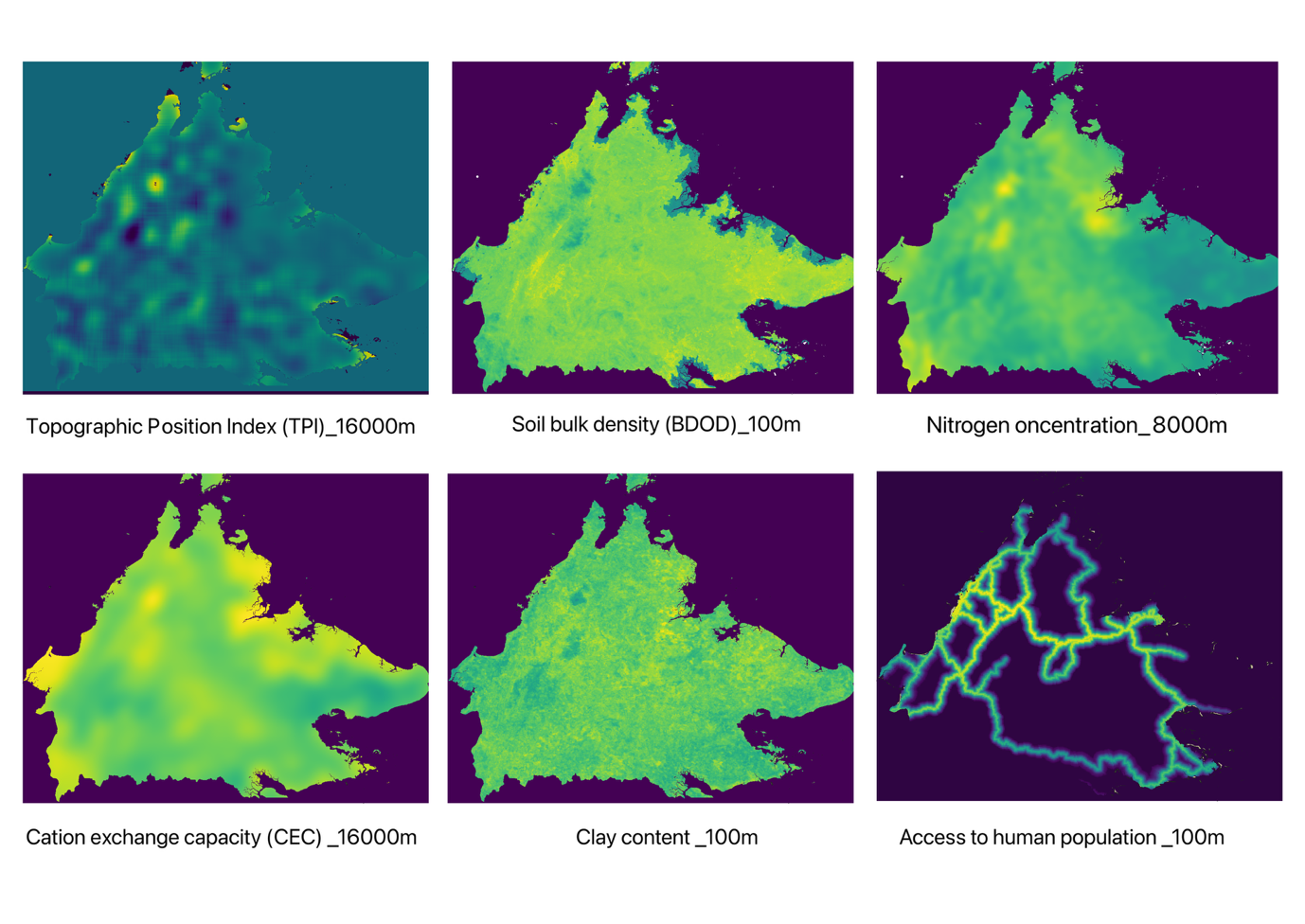


Figure S4: Plot of scale-optimised predictors used to fit final model of habitat suitability in Sabah.

Table S4: Final set of candidate variables and scale selected that were used to fit logistics regression model.

| Category | Variable | Scale selected (m) |
| --- | --- | --- |
| **Anthropogenic** | Secondary roads | 8000 |
|  | Accessibility Human Population (Hpop) | 100 |
| **Geomorphological** | Clay | 100 |
|  | Sand | 100 |
|  | Roughness | 4000 |
|  | Nitrogen | 8000 |
|  | Topographic Position Index (TPI) | 16000 |
|  | Cation exchange capacity(CEC) | 16000 |
|  | Soil organic carbon (SOC) | 16000 |
|  | Bulk density (BDOD) | 100 |
|  | Water | 4000 |
| **Biological** | Tree cover | 16000 |
|  | Degraded land cover | 100 |
|  | Deforested land cover | 16000 |


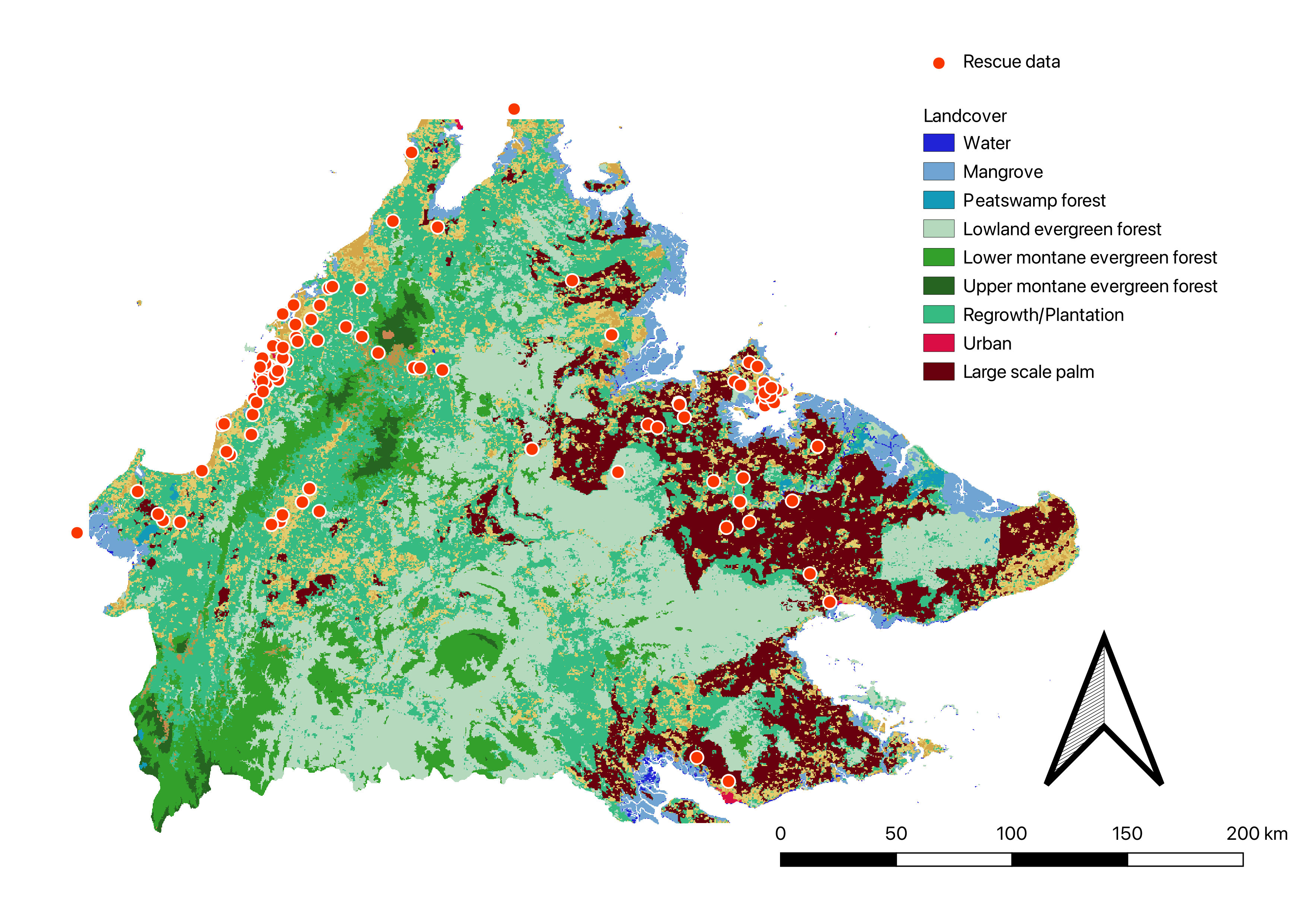


Figure S5: 149 rescue locations where Sunda pangolins were reported to the Sabah Wildlife Department between 2019-2024.

Table S5: List of predictors used in multi-scale model selection process. Only variables that included data for the year of survey was included to match value of a predictor with year data was collected.

| Category | Spatial predictor variable | Resolution(m^2^) | Year | Variable description |
| --- | --- | --- | --- | --- |
| Anthropogenic | Human Population Density | 1000 | 2000-2020 | Number of people per unit of land area |
|  | Global Modification Index | 1000 |  | human-induced landscape alterations |
|  | Roads Density | Shapefile | 2022 | Sabah road layer from (Panjang et al., 2024) |
|  | Protected Areas | Shapefile | 2022 | Boundaries of protected areas from WCMC and WDPA |
|  | Year since degradation | 30 | 2000-2021 | Categorical measure per pixel of degraded area |
|  | Forest loss | 30 | 2001-2021 | Year of forest loss per pixel |
|  | Tropical moist forest annual change | 30 | 1982-2023 | Categorical landcover level per pixel |
| Biological | Normalised difference vegetation index(NDVI) | 250 | 2000-2021 | Continuous density of vegetation area |
|  | Spectral variability vegetation index (SVVI) | 500 | 2000-2021 | Continuous index for classifying forest, secondary forest, and agriculture |
|  | Gross Primary Production | 500 | 2000-2021 | Total carbon fixation by vegetation |
|  | Continuous vegetation fields | 250 | 2000-2020 | Gradual vegetation cover representation |
|  | Canopy height | 30 | 2019 | Vertical measurement of forest or vegetation cover |
| Climatic | Land Surface Temperature | 1000 | 2000-2021 | Thermal radiation derived temperature |
|  | Mean Annual Temperature | 1000 | 1979-2013 | Average surface air temperature |
|  | Annual Precipitation | 1000 | 1979-2013 | Total yearly rainfall accumulation |
|  | Temperature seasonality | 1000 | 1979-2013 | Temperature variability |
|  | Precipitation Seasonality | 1000 | 1979-2013 | Rainfall distribution across seasons |
| Geomorphological | Elevation | 90 | 2000 | Height above sea level |
|  | Topographic Position Index | 90 | 2000 | Landform classification based on surrounding area |
|  | Compound Topographic Index | 90 | 2000 | Soil moisture potential based on topography |
|  | Roughness | 90 | 2000 | Surface irregularity |
|  | Soil Organic Carbon | 250 | 1960-2022 | Carbon stored in soil in form of organic matter |
|  | Bulk Density | 250 | 1960-2022 | Soil mass per unit volume |
|  | Cation Exchange Capacity | 250 | 1960-2022 | Soil’s ability to retain and exchange nutrients |
|  | Clay | 250 | 1960-2022 | Fine-textured soil component |
|  | Sand | 250 | 1960-2022 | Coarse-textured soil |
|  | Nitrogen | 250 | 1960-2022 | Soil nitrogen concentration |
|  | Water density | 30 | 1999-2020 | Surface water availability |
